# Benefits and challenges of a personal budget for people with mental health conditions or intellectual disability: A systematic review

**DOI:** 10.3389/fpsyt.2022.974621

**Published:** 2022-08-04

**Authors:** Martina Micai, Letizia Gila, Angela Caruso, Francesca Fulceri, Elisa Fontecedro, Giulio Castelpietra, Giovanna Romano, Mila Ferri, Maria Luisa Scattoni

**Affiliations:** ^1^Research Coordination and Support Service, Istituto Superiore di Sanità, Rome, Italy; ^2^Department of Mental Health, Friuli Centrale Healthcare Agency, Udine, Italy; ^3^Central Health Directorate, Friuli-Venezia Giulia Region, Trieste, Italy; ^4^Directorate General of Health Prevention, Ministry of Health, Rome, Italy; ^5^Emilia-Romagna Region, Bologna, Italy

**Keywords:** personal health budget, individual health budget, personalized care, health and social care policy and practice, mental disorder, intellectual disabilities

## Abstract

Personal budgets (PBs) may improve the lives of people with mental health conditions and people with intellectual disability (ID). However, a clear definition of PB, benefits, and challenges is still faded. This work aims to systematically review evidence on PB use in mental health and ID contexts, from both a qualitative and quantitative perspective, and summarize the recent research on interventions, outcomes, and cost-effectiveness of PBs in beneficiaries with mental health conditions and/or ID. The present systematic review is an update of the existing literature analyzed since 2013. We performed a systematic search strategy of articles using the bibliographic databases PubMed and PsycINFO. Six blinded authors screened the works for inclusion/exclusion criteria, and two blinded authors extracted the data. We performed a formal narrative synthesis of the findings from the selected works. A total of 9,800 publications were screened, and 29 were included. Improvement in responsibility and awareness, quality of life, independent living, paid work, clinical, psychological, and social domains, and everyday aspects of the users’ and their carers’ life have been observed in people with mental health conditions and/or ID. However, the PBs need to be less stressful and burdensome in their management for users, carers, and professionals. In addition, more quantitative research is needed to inform PBs’ policymakers.

**Systematic Review Registration:**

[www.crd.york.ac.uk/prospero/], identifier [CRD42020172607].

## Introduction

Personalization is a key element of the healthcare policy, as underlined by the World Health Organization (WHO) (1). It is built on a conception of a health system prioritizing human principles and rights, like equality, participation, self-determination, and non-discrimination. This means treating patients from a person-centered perspective, listening to their needs, and recognizing their capacities and freedom to choose their health. A person-centered approach should also tailor integrated services and goods to the needs of the individual with the goal of personal wellbeing (1). In this perspective, the delivery of the innovative intervention to set people at the center of their care and meet their identified health and wellbeing needs is a public health system’s strategic action.

Among organization and management models based on this healthcare approach, personal budget (PB) consists of a sum of money allocated to an individual with the specific aim of fulfilling personal health needs. It derives from an evolution of direct payment, introduced in the United Kingdom in 1997 for disabled people, and subsequently extended and implemented across all adult social care (2, 3). The budget can be directly given to the beneficiary, or it can be managed by an intermediary belonging to the social or the public healthcare, delegated to purchase the services according to the person’s needs (4, 5).

Although the adoption of PB for health and social care is a challenge for the healthcare system, implying a change from the traditional care model, several benefits have been identified. The patient is placed as a subject rather than an object of care and can be actively involved in deciding on how to spend the budget and which services may best suit their personal needs. PBs can promote autonomy and empowerment by giving the patient the chance to participate in care actively, and the beneficiaries may experience more control and flexibility over care providers. Moreover, PBs consider different kinds of essential parts of health, like housing, employment, education, relationships, cultural background, and greater integration between health and social care, finally enabling people to select services they need, may reduce costs, and improve care planning (6). However, concern has also been expressed on PBs. An inner risk of direct payment, for instance, can arise from spending money in ways that do not turn out to be effective or in abuse by PB holders (4). Other consequences may involve the lack of quality and assurance in employment conditions and the risk of creating an unregulated situation (7).

Personal budgets have been tested in different countries, including the United Kingdom, Belgium, Denmark, Italy, Finland, Austria, France, Sweden, Germany, Australia, and the United States, with different types of programs, varying from inclusion criteria related to commissioned services or the person’s degree of choice. Most programs aimed to promote autonomy and independent living, whereas others focused on the caregiver’s system. In Belgium, for instance, the aim was to reduce the use of expensive residential care, while in the United States it was a shortage of long-term staff. The target population also differed between countries. In Canada, PBs were directed to children with learning disabilities, whereas in the United Kingdom the focus was on the elderly and on subjects with long-term disabilities (8).

National and international PB’s experience mainly refers to users in charge of mental health services and their carers (5, 6, 9–11). This population has reported personal satisfaction related to the greater choice and motivation (12), greater participation in community life and supported employment related to self-directed initiatives (13), and overall better quality of life experienced with PBs’ programs (6). For example, the “Florida Self-Directed Care Program” offered to people with persistent mental illness resulted those participants rarely resorted to crisis stabilization units compared to non-participants (8). In a previous review, Webber et al. suggested positive outcomes regarding choice and control, impact on quality of life, service use, and cost-effectiveness for PB users in mental health. However, methodological limitations (i.e., different outcomes measures, limited follow-up periods, small sample size, and qualitative study design) limited the conclusions. Moreover, PB programs were implemented differently among countries, leading to additional difficulties in generalizing practices (14). Additional studies have been conducted in recent years, providing further evidence on the possible benefits of using PB in mental health settings. This is essential to inform current PB policies and practices with people with mental health conditions.

We believe that the inclusion, in the population benefiting of the PBs, of people with intellectual disability (ID) together with people with mental health conditions is important. Higher rate of mental illness in comparison with the general population is consistently observed in adults with ID (15, 16). A wide-ranging prevalence of 14–75% for clinically distinguishable mental illness in people with ID has been identified (17). ID is also considered among the leading psychiatric diagnoses and contributes, along with other mental disorders, to a major burden in terms of disability among young people (18). Mental health services should be prepared to meet the needs of people with ID.

In this study, thus, we aimed (a) to review the literature available from 2013 on PBs’ use in mental health contexts, from both a qualitative and quantitative perspective, and (b) to summarize the recent evidence on interventions, outcomes, and cost-effectiveness of PBs in beneficiaries with mental health conditions and/or with ID.

## Materials and methods

### Search strategy

First, we interrogated PROSPERO^1^ to search for ongoing systematic reviews. Since no systematic reviews were scheduled, our protocol was registered with PROSPERO: CRD42020172607. The present systematic review followed the guidelines of the Preferred Reporting Items for Systematic Reviews and Meta-Analyses (PRISMA) [Supplementary Figure 1; (19)]. We performed a systematic search strategy of articles indexed from 01 April 2013 to 15 September 2021 using the bibliographic databases PubMed and PsycINFO. We choose to start our search strategy in April 2013 because the latest systematic review on the field ended its search strategy in that period (14). The search strategy focused on Population, Intervention, Comparison, and Outcomes (PICO) domains. Population: PB for people with mental health conditions and/or people with ID. Intervention: terms related to the PB; Comparison: not applicable; Outcome: desired and undesirable effects of PB. We developed the search strategy using a combination of MeSH (Medical Subject Headings) and terms to capture the available literature on the topic. The present search strategy was used for PubMed database, including planned limits, such that it could be repeated, and was prepared and adapted using appropriate syntax for PsycINFO database. When available, search filters were applied to limit the search to “Humans.” Language restriction to English has been applied. This search strategy was peer-reviewed by experts in the field. Details of the search strategy are presented in Supplementary Table 1. Moreover, the reference lists from identified studies were scanned to identify any other relevant studies. We contacted experts in the field to determine whether there are any ongoing trials or unpublished results in this area.

### Selection process

**FIGURE 1:**
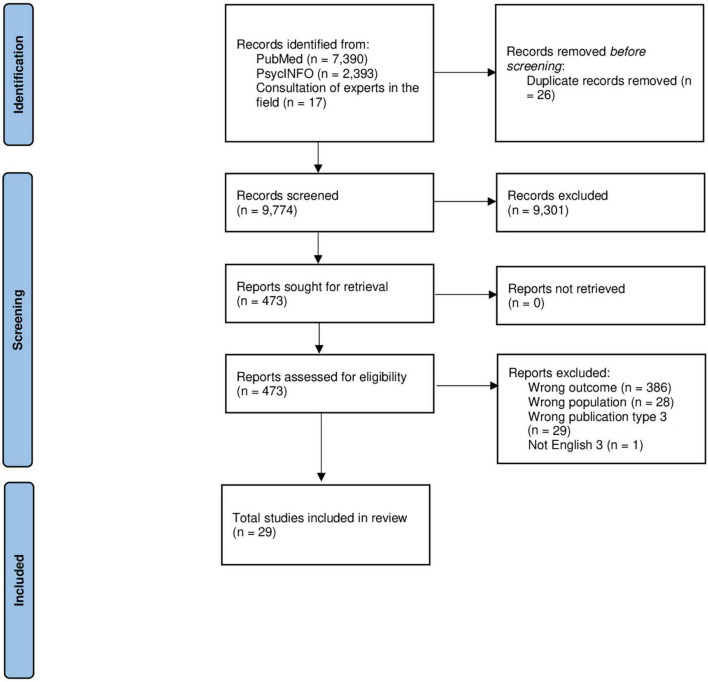
Flow chart of the literature selection process.

The articles/reviews detected through the search strategy were collected in the Systematic Review Rayyan QCRI application (20), which also supported the authors in excluding duplicates. Six blinded authors screened the works for inclusion and exclusion criteria (LG, MM, AC, FF, EF, and GC), and two blinded authors extracted the data (LG and MM). We included quantitative and qualitative studies that report methods and models of PB for people with mental health conditions. We excluded works not in English and published before April 2013. The works that did not meet the inclusion criteria based on the titles and abstracts were excluded by at least two independent authors. Works were checked in their full text by at least two independent authors. Conflicts were discussed between two authors, and if needed, the consultation of third independent author was requested. Figure 1 illustrates the flow chart of the literature selection process.

### Data extraction and synthesis

The data of the works that passed the selection process were extracted in a data extraction Excel form (available on request). To ensure consistency across authors, we conducted calibration exercises before starting to extract the data. Two independent authors (LG and MM) collected the general information of the publication: first author, year of publication, country where was conducted the study, funding sources, and potential conflict of interests. We collected the study’s design, description and sample size of the intervention group, description and sample size of the control group(s), age in years of the population (mean, SD, and range), percentage of females, name of the intervention, instrument used to measure the outcomes, length of the follow-up (if present), length of the budget, item purchased (Table 1), and summary of findings (Table 2). The data from the included full texts were extracted and independently cross-checked by two authors (LG and MM). We performed a formal narrative synthesis of the findings from the selected works.

**TABLE 1:** Summary of the description of the included works on personal budget for people with mental health conditions.

Study		Population	Intervention	Outcome measures	Additional data
References (country)	Design	(1) Sample size intervention group (description, *n*); control group (description, *n*) (2) Age in years (mean ± SD, range) (3) % females			(1) Length of follow-up (2) Length of budget (3) Item purchased
Adinolfi et al. (30) (Italy)	Qualitative	(1) 43 service users (severe mental disorders) (2) 58.7 ± 12 (3) 32.5%	HB	HoNOS; perceived quality of services; cost savings*^a^*	(1) 1 year (2) 1 year (3) Health status, well-being, satisfaction with care and cost savings
Bowdoin et al. (26) (United States)	Quasi experimental	(1) 1,466 service users in patient-centered medical home (PCMH) (mental illness); 4,709 individuals in non-PCMH usual source of care (USC); 733 individuals in no USC (2) PCMH: range = 18–34: 27.3%; 35–49: 36.5%; 50–64: 36.2% Non-PCMH USC: 18–34: 27.3%; 35–49: 34.2%; 50–64: 38.6% No USC:18–34: 57.7%; 35–49: 29.3%; 50–64: 16.0% (3) PCMH: 70.9% Non-PCMH USC: 67.6% No USC: 51.9%	Patient-centered medical home	Self-reported data	(1) NA (2) 2 years (2) Healthcare services utilization and expenditures
Cook et al. (31) (United States)	Quantitative	(1) 114 service users (serious mental illness); 102 care as usual (serious mental illness) (2) Self-directing: 41.6 ± 10.0 service as usual: 41.6 ± 9.5 (3) Self-directing: 62% service as usual: 67%	Self-directed care	Recovery Assessment Scale; subscale of the Empowerment Scale; Coping Mastery Scale; Perceived Autonomy Support Scale; Brief Symptom Inventory’s Global Severity Index; employment and education/training*^c^*; Client Satisfaction Questionnaire	(1) 2 years (2) NA (3) Transportation, communication, medical care, residential, and health and wellness needs
Croft and Parish (46) (United States)	Qualitative	(1) 30 service users (physical and developmental disabilities, traumatic brain injury) (2) NA (3) 56.6%	Person centered planning	Study-specific in-depth interview guide	(1) NA (2) Unclear (3) Support to employment, housing, and hobbies
Croft et al. (27) (United States)	Quasi-experimental	(1) 271 self-directing service users (physical and developmental disabilities, traumatic brain injury); 1,099 non-self-directing individuals (2) Self-directing: 51.99 ± 10.28 Non-self-directing: 51.78 ± 11.10 (3) Self-directing: 72% Non-self-directing: 72%	Individual budget	Employment; independent housing^b^	(1) Program A: 4.8 years; program B: 3 years (2) Unclear (3) Employment; independent housing
Croft et al. (28) (United States)	Quasi experimental	(1) 94 service users (serious mental illness); 529 care as usual (serious mental illness) (2) MHATR: 42.38 ± 10.11 Non-MHATR: 42.85 ± 12.30 (3) MHATR: 38.2% Non-MHATR: 38.3%	Mental Health Access to Recovery or MHATR	Service utilization data^d^; GAF	(1) Unclear (2) Unclear (3) Transportation, dental care, emergency housing, wellness and self-care, and special needs
Croft et al. (29) (United States)	Quasi experimental	(1) 45 service users (schizophrenia, major depression, bipolar disorder) (2) 51.5 ± 9.8 (3) 71.1%	Self-directed care	Pre- and post-program Medicaid managed care claims data for CRIF-SDC II	(1) NA (2) NA (3) Routines and manage stress, mobility/transportation, self-care, domestic activities, education, employment/volunteering, social and community life
Fontecedro et al. (49) (Italy)	Cross-sectional	(1) 67 service users Individual HB beneficiaries (psychoactive substances, psychotic, affective, personality and other psychiatric disorders); 61 individuals in care as usual (2) Individual HB: range = 20–29: 17.9%; 30–39: 11.9%; 40–49: 23.9%;50–59: 20.9%; 60–69: 17.9%; ≥70:7.5% Care as usual: 20–29: 4.9%; 30–39: 14.7%; 40–49: 14.7%;50–59: 31.1%; 60–69: 19.7%; ≥70:14.7% (3) Individual HB: 37.3% Care as usual: 62.7%	Individual HB	Clinical variables, type of Individual HB, HoNOS	(1) NA (2) 15 months (3) NA
Hamilton et al. (45) (United Kingdom)	Qualitative	(1) 28 professionals (mental health practitioners) (2) NA (3) 57.1%	Personalization of the care	Semi-structured guide interview, developed from existing literature and findings from earlier fieldwork	(1) NA (2) Unclear (3) NA
Hamilton et al. (50) (United Kingdom)	Qualitative	(1) 12 carers (schizophrenia/related psychosis, bipolar disorder, depression, multiple diagnoses) (2) Range = 21–71 (3) 66.6%	PB	Study specific in-depth qualitative interviews	(1) NA (2) Unclear (3) NA
Hamilton et al. (44) (United Kingdom)	Qualitative	(1) 52 Service users (schizophrenia and related psychotic disorders, bipolar disorder, depression, personality disorder, other, multiple diagnoses); 48 professionals (social workers, occupational therapists, and community psychiatric nurses) (2) NA (3) Service users: 61.5% Professionals: 33.3%	PB	Study specific semi-structured topic guide interview	(1) NA (2) Unclear (3) NA
Harry et al. (48) (United States)	Qualitative	(1) 11 service users (intellectual disability) (2) Mean = 29 ± 3.28, 23–34 (3) 45.4%	Cash and counseling-based self-directed services program	Study-specific open-ended, semi-structured interview guide	(1) NA (2) Unclear (3) Services and supports for activities of daily living
Hitchen et al. (54) (United Kingdom)	Qualitative	(1) 11 service users; 21 carers; 12 professionals (local authority, trust staff, managers, third-sector representative) (2) NA (3) NA	PB	Focus groups	(1) NA (2) Unclear (3) NA
Kogan et al. (32) (United States)	Qualitative	(1) 516 service users in Patient Self-Directed Care (serious mental illness), 713 in Provider- Supported Integrated Care (2) ≥21 (3) NA	Behavioral Health Home Intervention Arm	Self-report data; existing health service claims data; interviews	(1) 2 years (2) Every 6 months (3) Health and wellness
Larkin (43) (United Kingdom)	Qualitative	(1) 23 careers (2) Range = 30–65 (3) 69.5%	PB	Study specific semi-structured in-depth interviews	(1) NA (2) Unclear (3) NA
Larsen et al. (52) (United Kingdom)	Qualitative	(1) 47 service users (schizophrenia and other psychotic disorders, bipolar disorder, depression, personality disorder, other, multiple diagnoses) (2) 46, range = 21–71 (3) 61.7%	PB	Study specific in-depth semi-structured interviews	(1) NA (2) Unclear (3) Wellbeing; social participation
Leuci et al. (33) (Italy)	Cohort	(1) 49 service users in PHB in association with pharmacological therapy (first-episode psychosis); 55 in pharmacological therapy (2) PHB + pharmacological therapy: 26.08 ± 6.29 (3) Pharmacological therapy: 30.56 ± 8.78 PHB + pharmacological therapy: 26.5% Pharmacological therapy: 43.6%	PHB in association with pharmacological therapy	BPRS; GAF; HoNOS	(1) 2 years (2) 2 years (3) Housing, employment and/or social participation
Mitchell et al. (47) (United Kingdom)	Qualitative	(1) 47 professionals (practitioners from older people and learning disability) (2) NA (3) NA	PB	Focus groups	(1) NA (2) Unclear (3) NA
Norrie et al. (39) (United Kingdom)	Qualitative	(1) 131 professionals (105 personal assistants and 26 key informants) (2) Personal assistants: 44.8, 20–70 (3) Personal assistants: 87%	PB	Structured interviews with open and closed format questions	(1) NA (2) Unclear (3) NA
Pelizza et al. (34) (Italy)	Cohort	(1) 49 Service users (mental health conditions) (2) 26.08 ± 6.29 (3) 85.7%	PHB	BPRS – version 4.0; GAF scale 11; HoNOS	(1) 2 years (2) 2 years (3) Housing, employment and/or social participation
Pelizza et al. (35) (Italy)	Cohort	(1) 137 service users (schizophrenia or other psychotic disorder, bipolar disorder with psychotic features, major depressive disorder with psychotic features) (2) 32.74 ± 11.15 (3) 68.7%	PHB	BPRS – version 4.0; GAF scale 11; HoNOS	(1) 2 years (2) 2 years (3) Housing, employment and/or social participation
Peterson et al. (40) (Australia)	Qualitative	16 service users (depressive and/or substance induced psychotic disorders, and single incidences of anxiety, stress, and delusional disorders) (4) 46, range 40–64 (5) 56.2%	Shared management, person-centered and self-directed (SPS) service	Study specific interview	(1) NA (2) Unclear (2) Training, equipment, social participation
Ridente and Mezzina (36) (Italy)	Qualitative	(1) 66 service users (people in residential facilities) (2) NA (3) NA	Supported housing	Not specified	(1) 10 years (2) Unclear (3) Housing, employment, social relationships
Snethen et al. (53) (United States)	Cross-sectional	(1) 60 service users (non-acute serious mental illness) (2) 44.9, 18–65 (3) 72%	PB	Section on activities and participation of the WHO ICF model	(1) NA (2) Unclear (3) Non-traditional goods and services
Spaulding-Givens et al. (41) (United States)	Qualitative	(1) 18 service users (mood disorder, substance abuse) (2) 54 ± 11.8, 29–70 (3) 61.1%	Individual budget (self-directed care)	Study-specific interview	(1) NA (2) Unclear (3) Basic needs, mental health, physical fitness, education and technology, and miscellaneous supplies
Tew et al. (51) (United Kingdom)	Qualitative	(1) 53 service users (schizophrenia and related psychotic disorders, bipolar disorder, depression, personality disorder, other, multiple diagnoses) (2) 44 (3) 62%	PB	Study specific in-depth qualitative semi-structured topic guide interviews	(1) NA (2) Unclear (3) NA
Thomas et al. (37) (United States)	Qualitative	(1) 45 service users (schizophrenia, major depression, bipolar disorder) (2) 45.48 ± 10.80 (3) 71%	Self-directed care	Study-specific semi-structured interview	(1) 2 years (2) Unclear (3) Non-traditional goods or services
Welch et al. (42) (United Kingdom)	Qualitative	(1) 10 professionals (organizational representatives) (2) NA (3) NA	PHB	Study-specific semi-structured interview	(1) NA (2) Unclear (3) NA
Williams and Porter (38) (United Kingdom)	Qualitative	(1) 9 service users (intellectual disability) (2) range = 19–62 (3) 55.5%	PB	Study-specific semi-structured qualitative interviews	(1) NA (2) Unclear (3) Unclear

NA, not applicable for the item; HB, health budget; PB, personal budget; PHB, personal health budget; HoNOS, Health of the Nation Outcome Scale; GAF, Global Assessment of Functioning Scale; CRIF-SDC II, Consumer Recovery Investment Fund-Self-Directed Care; BPRS, Brief Psychiatric Rating Scale; WHO-ICF, World Health Organization’s International Classification of Function, Disability, and Health.

^a^Assessment of perceived quality of services: qualitative semi-structured questionnaire (55); assessment of cost savings: comparison of real expenditures associated with the health budget initiative.

^b^Employment assessment: number of days worked housing independence assessment: transition from dependent housing or homelessness to living independently, or maintenance of independent housing status.

^c^Employment assessment: the U.S. Department of Labor’s definition of any work at all for pay or profit during a reference week; education training assessment: the U.S. Department of Education’s definition of formal education as instruction provided in a system of schools, colleges, universities, and other formal education institutions.

^d^Service utilization data: treatment services, rehabilitation services, residential services, and emergency services.

### Quality assessment of the evidence

Two independent authors (MM and LG) assessed risk of bias in the selected studies. Twenty eligible qualitative works were evaluated through 10-questions Critical Appraisal Skills Programme (21) Qualitative Study Checklist; two cross-sectional studies were evaluated using the Joanna Briggs Institute (JBI) Checklist for Analytical Cross-Sectional Studies (22); three cohort studies were evaluated using the Critical Appraisal Skills Programme (23); Critical Appraisal Skills Programme (24) and four quasi-experimental studies were evaluated using the JBI Critical Appraisal Checklist for Quasi-Experimental Studies (non-randomized experimental studies) (25). Any disagreements were solved in conjunction with a third author (AC). Supplementary Tables 2–5 summarize the risk-of-bias assessment results.

**TABLE 2:** Findings’ summary of the included works on PHB for people with mental health conditions.

Adinolfi et al. (30) (Italy)	HB led to significant cost savings, mostly associated with the reduction of the cases of institutionalization and the higher appropriateness of health care services. HB let to more suitable health treatment, reducing redundancies and omissions. HB led to improvement in problems related to alcohol and/or drug addiction; cognitive, physical or disability problems; problems associated with hallucinations and delusions, with depressed mood; mental and behavioral problems; problems with relationships, with activities of daily living, with living conditions, and with occupation and activities. Patients and informal caregivers expressed medium-high levels of satisfaction with the HB program.
Bowdoin et al. (26) (United States)	No statistically significant differences between participants who received patient-centered medical home (PCMH) care and participants who received non-PCMH care or usual care in terms service utilization, cost, or expenditures.
Cook et al. (31) (United States)	The budget-neutral self-directed care model achieved superior client outcomes and greater satisfaction with mental health care, compared with services as usual. Self-directed care users compared to the control group showed greater improvement over time in recovery, self-esteem, coping mastery, autonomy support, somatic symptoms, employment, and education. The costs of self-directed care users were lower, comparing with the control group. Self-directed care users reported higher satisfaction with mental health services.
Croft and Parish (46) (United States)	The program participation helped the majority of responders to meet basic material needs that had been impeding them from achieving or setting personal goals. Six interviewees expressed confusion and frustration at what they saw as inconsistent policies, particularly for purchases that address basic needs like rent, transportation, and household goods.
Croft et al. (27) (United States)	Self-directing participants were more likely to improve or maintain engagement in paid work (small effect size) and independent housing (small effect size).
Croft et al. (28) (United States)	Self-directing users showed greater increases in outpatient and rehabilitation services compared to the non-self-directing group, in terms of hours of rehabilitation services. No differences in residential days or emergency service hours between groups.
Croft et al. (29) (United States)	No difference in the percentage of individuals who used at least one service in each service category before and after participation to the program. Lower median standardized monthly mental health clinical outpatient costs compared to the past by a model of pre-post examination. No differences in the total service costs before and after program participation.
Fontecedro et al. (49) (Italy)	The Individual Health Budget (IHB) was used in patients with severe clinical and social problems. The beneficiaries were at higher risk compared to controls of severe problems with regard to aggressive or agitated behaviors, hallucinations and delusions, and impairment in everyday life activities. The risk of hospitalization in the IHB sample compared to controls was 1.4-fold higher, regardless of diagnosis. The clinical and functional impairment (HoNOS total scores) did not differ between IHB and controls.
Hamilton et al. (45) (United Kingdom)	Most of the practitioners interviewed felt that they had already always worked in patient-centered ways. Most of all reported to be more affiliated with the dominant medical model, rather than the person-centered model. Some mental health nurses expressed that the PB is perceived as a distraction from the work of nursing. Other nurses argued that specific skills that characterized their profession were being side-lined to engage in implementing PBs. Several social workers described their priorities in terms of a medical model of mental health, requiring treatment first and social support later. They reported the perception of insufficient time to engage with the process of PBs, perceiving it as bureaucratic, complicated and time consuming. Social workers identified the voluntary or third sector as a potential resource for PB. Occupational therapists described PBs as fitting more closely with occupational therapy practice. They often found personalization to be an opportunity to reclaim their focus away from the medical model and back to the traditional goals of occupational therapy.
Hamilton et al. (50) (United Kingdom)	Carers were commonly involved in decisions made through assessment, support planning and reviews the PB. Criticisms of the PB by carers: the process for accessing and reviewing PBs was not well designed for people with severe mental health conditions to manage: the “paperwork” was stressful; carers felt the role to protect the service user from mental health systems and practitioners who were not making decisions in their best interests; carers perceived that PB funding was reduced because of practitioners’ assumptions about carers’ willingness and ability to provide support; conflicts with staff around appropriate involvement in decision-making. Parents often felt the need to help the service user to be heard in assessment and support planning meetings. Partners, when considering a PB, felt treated more as a unit.
Hamilton et al. (44) (United Kingdom)	Opportunities of the PB perceived by user services and staff: users’ power and control, collaboration with staff, and quality and continuity of the professional relationship. Challenges of the PB perceived by user services: few of them felt unable to manage the budget and saw the staff as an obstruction to the PB. Challenges of the PB perceived by staff: few of them were reluctant to engage the PB. The process of reaching decisions of local authority led them to feel a sense of distance that limited their power of influence.
Harry et al. (48) (United States)	All participants were satisfied about the Cash and Counseling-based self-directed services program and perceived their personal care need met. All participants trusted their caregiver(s). Two women talked about how the program reduced financial stress on the family and young adult. Five representatives described financial concerns with the monthly allowance amount left after paying attendants and confusion about program rules.
Hitchen et al. (54) (United Kingdom)	Users, carers, and staff perceived the need for cultural change, PBs’ effect on outcomes, and service-users’ capacity to manage these responsibilities.
Kogan et al. (32) (United States)	The use of historical claims data can lead to an overestimation of eligible participants and, subsequently, a reduced study sample and an imbalance between intervention arms. Inclusion of multiple data sources in study design can improve data completeness. The use of a “train-the-trainer” model, “wellness champions,” and the use of a Learning Collaborative approach may help in overcoming training and intervention fidelity challenges (i.e., geographic dispersion of rural provider sites and staff turnover). Stakeholder engagement may mitigate these challenges critical to study progress.
Larkin (43) (United Kingdom)	Perceived positive effects of PB by carers: enhancement of the carer–service user relationship, feeling happier, healthier and having more control over their lives. Perceived negative effects of PB by carers: less involvement in the service user’s care, perception of the administration of the PB as stressful.
Larsen et al. (52) (United Kingdom)	Most participants identified positive outcomes from using PBs (e.g., mental health and wellbeing, social participation and relationships, and confidence). Some participants needed more support to identify goals and make use of the PB to take a more active part in the society.
Leuci et al. (33) (Italy)	Significant effect of time on functioning and all the psychopathological and clinical variables in patients enrolled and not enrolled in PHB across the 2 years of follow-up. At the end of the 2 years follow-up, patients enrolled in the PHB intervention reported higher improvements in negative symptoms, disorganization, and activation dimension (including manic features and hyperactivity) and in HoNOS “Impairment” subscale (combining cognitive and disability problems). In the time between T1 and T2, patients enrolled in the PHB showed a further significant decrease in positive symptoms, activation, and affective dimension (including depressive characteristics, suicidality, and anxiety), improvements in functioning, and in HoNOS “Impairment,” “Psychiatric Symptoms,” and “Behavioural Problems” subscales. Significant “time x group” interaction effects in BPRS “Disorganization,” HoNOS “Psychiatric Symptoms,” and GAF scores in patients enrolled in the PHB, suggesting an additional improvement both in symptoms and in functioning for those receiving the PHB intervention model.
Mitchell et al. (47) (United Kingdom)	All practitioners’ focus groups reported that their authorities recognized the importance of involving carers in the service user personalization processes. Involvement in support planning of practitioners and carers was considered necessary. Moreover, a good support plan for service users was perceived as having indirect benefits for carers. Carers were reported to be often involved in the managing of the PBs. Staff reported that conflicts on service users’ abilities and support needs between staff and service users and carers were most likely to arise during assessment and support planning of the PB. In the management of conflicts were considered important “good” social work skills and practitioner sensitivity.
Norrie et al. (39) (United Kingdom)	The 64% of personal assistants saw their current roles as congruent with PHBs, were willing to engage with PHBs and undertake health-related tasks. The 74% of personal assistants perceived the need of additional training if enacting PHB. Key informants perceived the development of HBs as complex.
Pelizza et al. (34) (Italy)	PHB approach within an “Early Intervention in Psychosis” program showed a significant effect of time on all Health of Nation Outcome Scales (HoNOS), Brief Psychiatric Rating Scale (BPRS) and Global Assessment of Functioning (GAF) scores along the 2 years of follow-up. PHB multiaxial intervention was associated with longitudinal decrease in BPRS “Negative Symptoms,” HoNOS “Behavioral Problems,” and “Social Problems” subscores.
Pelizza et al. (35) (Italy)	PHB approach showed a significant decrease in all GAF scale, HoNOS and BPRS scores along the 2 years of follow-up. PHB multiaxial intervention was associated with longitudinal decrease in BPRS “Negative Symptoms,” and HoNOS “Social Problems” subscores.
Peterson et al. (40) (Australia)	The three over-arching categories impacting the lived experiences of shared management, person-centered and self-directed (SPS) consumers individuated by the authors were: (1) Access to individualized funds enabled practical and psychological benefits to consumers; (2) Consistent contact in shared management and person-centered relationships enhanced the provision of timely and meaningful staff support to consumers; (3) High quality shared management and person-centered relationships with staff and the opportunity to self-direct services enabled consumers’ change and growth.
Ridente and Mezzina et al. (36) (Italy)	The individual HB method boosted the shift toward personalized supported housing for people with severe mental health conditions and complex problems. For people, the use of HB method resulted in increased personal autonomy and higher personalization of interventions. For services, the use of HB method brought about significant changes is the way resources were used and in the personalized intervention approach within local Mental Health Department teams (transparency, clarity on spending decisions, and increased awareness of the importance of a rational use of resources based on an adequate turnover of projects). Regarding housing, the number and type of community organizations involved in the co-management of HBs has increased significantly by a closer collaboration and synergy among different third sector agencies.
Snethen et al. (53) (United States)	The majority of people with serious mental illness can identify a number of goods or services not traditionally available through Medicaid that would facilitate their mental health. Fewer than 10% of requests across all participants were categorized within unique World Health Organization’s International Classification of Function, Disability, and Health (ICF) codes. Needs changed depending on the diagnosis and were consistent with the ICF diagnostic core sets.
Spaulding-Givens et al. (41) (United States)	Users reported that individualized budgeting and purchasing contributed to their mental wellness, stability, and self-esteem; enhanced their control over service choices, and provided some material relief in ongoing struggles with chronic poverty. Several participants reported that coaches played an important role in supporting participants’ self-direction by mitigating potential purchasing barriers (lack of clarity and flexibility in purchasing guidelines, technicalities in purchasing procedures, perceived disconnect between purchases and goals, and participants’ struggles with symptoms). Flexible spending guidelines and streamlined approval processes are needed; overly proscriptive policies may undermine participants’ self-determination.
Tew et al. (51) (United Kingdom)	Some participants did not find it easy to adjust to the opportunity to think and take responsibility for themselves. Participants highlighted how practitioners adopting a co-productive or coaching style of working could help them broaden their horizons. Several participants declared that an access to economic capital enabled them to have better quality of day-to-day life. Others saw their budget as a mechanism for organizing personal support in terms of how they were living their lives. The peer supporting has an emotional significance for the persons involved.
Thomas et al. (37) (United States)	The majority of participants reported experiencing greater choice and control in the selection of services for supporting recovery goals, as a result of their participation in self-direct care intervention. Competence, autonomy, and relatedness were well connected to participant’s experiences of increased choice.
Welch et al. (42) (United Kingdom)	PHB-holders and front-line staff perceived the following opportunities: increase in choice and control; improvement in relationships between budget-holders and their care staff; increase in power to service providers and commissioners. PHB-holders and front-line staff perceived the following operational challenges: “when” to offer a PHB as often clients were referred at “crisis point”; what support could be purchased from the PHB, and it was thought that more guidance was required; the budget was held by the commissioner; risk, accountability, and safety. The importance of strong leadership was viewed as a crucial factor by all staff groups.
Williams and Porter et al. (38) (United Kingdom)	All the participants relied on praise and encouragement, and when this was not forthcoming, they became anxious or unsure of themselves. People members of self-advocacy groups helped other people with intellectual disabilities and contributed toward society through voluntary work or within their family. Although relationships were key to participants, they played out very differently in the various contexts of their lives. Despite the lack of real engagement with the PB processes, people clearly valued their own choices. What was needed by all participants was a more personalized approach which would understand the detail of their sense of identity, the people in their lives, and their day-to-day living, for them to build a continued sense of control.

## Results

The search strategy provided 9,800 works (PubMed, *n* = 7,390; PsycINFO, *n* = 2,393, and 17 consulting experts in the field). One author (MM) removed 26 duplicates. A total of 9,774 works were screened for inclusion and exclusion criteria. Based on the titles and abstracts screening, 9,301 not pertinent works were excluded. The remaining 473 works were checked in their full text. Studies not exploring PB (*n* = 386, wrong outcome) or exploring PB outside the mental health context and/or ID, including dementia, were excluded (*n* = 28, wrong population). In addition, 29 works were excluded because of reviews, case reports, comments, editorials, and letters (wrong publication type). Finally, one study was excluded because not in English. Conflicts were solved between authors, and for 69 works, the consultation of a third independent author was required. Finally, we evaluated 29 works as eligible for the data extraction process. Figure 1 provides the process of records’ identification and screening, and the eligibility and inclusion actions (19). Supplementary Appendix lists the reference of the 29 works included in the systematic review.

A meta-analysis of the extracted data was not possible since data of the systematic reviews were mostly qualitative and heterogeneous in the description of the PBs.

### Risk-of-bias assessment

Regarding the 20 qualitative studies, the case 2A intra-class correlation between reviewers was high (0.94; 95% CI = 0.80–0.98). This value indicates excellent reliability. Risk-of-bias overall rating ranged from 2 to 9 (mean = 5.70; SD = 2.53). The most common weaknesses were observed for the item number 3 (appropriate research design) for 14 studies, number 6 (researcher reflexivity) for 19 studies, number 8 (appropriate data analysis) for 13 studies, and number 9 (clear statement of findings) for 11 studies. Risk-of-bias ratings of the included qualitative studies are reported in Supplementary Table 2.

Regarding the two cross-sectional studies, the case 2A intra-class correlation between reviewers was good (0.81; 95% CI = 0.49–0.93). This value indicates excellent reliability. Risk-of-bias overall rating ranged from 5 to 7 (mean = 6.00; SD = 0.80). In all studies, the weaknesses were observed for the item number 6 (statement of strategies to deal with confounding factors). Risk-of-bias ratings of the included qualitative studies are reported in Supplementary Table 3.

Regarding the three cohort studies, the case 2A intra-class correlation between reviewers was 1 (absolute agreement). The weaknesses observed in all studies were for the domain numbers 5b (consideration of confounding factors in design and/or analysis), 6a (follow-up complete), 6b (follow-up long enough), and 7 (strong exposure and outcome relation). Risk-of-bias ratings of the included qualitative studies are reported in Supplementary Table 4.

Finally, regarding the four quasi-experimental studies, the case 2A intra-class correlation between reviewers was 1 (absolute agreement). Risk-of-bias overall rating ranged from 3 to 8 (mean = 6.00; SD = 2.20). In all studies, we observed weaknesses for the domain number 3 (participants included in any comparisons received similar treatment/care, exposure/intervention). Risk-of-bias ratings of the included qualitative studies are reported in Supplementary Table 5.

### Description of the studies

First author, study’s year, country where was conducted the study, study’s design, description and sample size of the intervention group, description and sample size of the control group(s), age in years of the population (mean, SD, and range), percentage of females, name of the intervention, instrument used to measure the outcomes, length of the follow-up (if present), length of the budget, and item purchased are displayed in Table 1. Table 2 shows the findings’ summary of the included studies.

We found that 18 studies declared no conflict of interests, while 11 did not provide this information. Eleven studies were conducted in the United Kingdom, 11 in the United States, 6 in Italy, and 1 in Australia. Four quasi-experimental studies were found (26–29). Nine studies reported follow-up data (27, 30–37). A total of 11,541 people participated in the 29 selected studies (range = 9; (38) – 6,908; (26); in studies conducted by the same authors, the participants may overlap). The mean ages of samples ranged from 26.08 (33, 34) to 58.7 (30) years. The proportion of females ranged from 26% (33) to 87% (39). Studies evaluated PB (*n* = 9), personal health budgets (*n* = 4), health budget (*n* = 1), individual budget (*n* = 1), self-directed care (*n* = 3), individual health budget (*n* = 2), patient-centered medical home (PCMH) (*n* = 1), person-centered planning (*n* = 1), Mental Health Access to Recovery or MHATR (*n* = 1), personalization of the care (*n* = 1), Cash and Counseling-based self-directed services program (*n* = 1), behavioral health home intervention arm (*n* = 1), shared management (*n* = 1), person-centered and self-directed (SPS) service (*n* = 1), and supported housing (*n* = 1), as defined by the authors, to define PB, which exist different acronyms (e.g., PB, PHB, and HB). We maintained definitions and acronyms used in each original work.

Eight studies [only two with quantitative data, (30, 31)] contained validated measures (e.g., Health of the Nation Outcome Scales, Brief Psychiatric Rating Scale, Global Assessment of Functioning, and World Health Organization’s International Classification of Functioning, Disability, and Health).

We maintained the domains’ classification given by Webber et al. (14) (i.e., choice and control of care and support; impact on life; service use; economic evaluations) and, for clarity seek, four authors classified the studies based on further subdomains (for choice and control of care and support: patient center-care, stakeholder engagement, involvement of carers or staff, timely and suitable access to treatment, phenotype assessment of HB patient, satisfaction of users, challenges of users, carers, and professionals; for impact on life: clinical improvement, quality of life, improvement in everyday life activities, engagement in work/independent housing, and frequency and quality of service use; for service use: frequency and quality of service use and lack of transitional support).

#### Choice and control of care and support

##### Patient center-care

The involvement of patients in the process of managing their care led to significant outcomes in terms of health status improvement, personalization of interventions, user satisfaction, increase in autonomy, self-esteem, competence, coping mastery, autonomy support, choice and control, control over service choices, and cost savings (30, 31, 36, 37, 40–42). Also, personalization can have positive outcomes for carers regarding their control over their daily lives, quality of life, health, and wellbeing (43). Hamilton et al. (44) showed that the staff also perceived users’ power and control. Most of the practitioners interviewed by Hamilton et al. (45) felt that they had already worked in patient-centered ways. However, most of all reported being more affiliated with the dominant medical model than the person-centered model. The Croft and Parish (46) and Spaulding-Givens et al. (41) studies provided insights for helping patients achieve or set their personal goals through the support in meeting their basic material needs.

##### Stakeholder engagement

Kogan et al. (32) affirmed that stakeholder-driven investigations might mitigate patient-centered comparative effectiveness research challenges.

##### Involvement of carers or staff

Peterson et al. (40) and Welch et al. (42) highlighted the importance of strong leadership among staff groups and the possibility of increasing power to service providers and commissioners. In the Norrie et al. (39), 64% of personal assistants saw their current roles as congruent with PHBs, were willing to engage with PHBs and undertake health-related tasks. At the same time, 74% of personal assistants perceived the need for additional training if enacting PHB (39). The staff interviewed by Mitchell et al. (47) reported that conflicts on service users’ abilities and support needs between staff and service users and carers were most likely to arise during assessment and support planning of the PB. In the management of conflicts were considered important “good” social work skills and practitioner sensitivity (47).

Mitchell et al. (47) showed that all practitioners’ focus groups reported that their authorities recognized the importance of involving carers in the service user personalization processes and support planning. Moreover, a good support plan for service users was perceived as having indirect benefits for carers. Carers were reported to be often involved in the managing of the PBs (42). Finally, Harry et al. (48) showed that all participants trusted their caregiver(s).

##### Timely and suitable access to treatment

Patients with severe and persistent mental disorders that benefit of the person-centered services had timely allocation of funds (40), suitable health treatment (30), and greater increases in outpatient and rehabilitation services compared to the non-self-directing group, in terms of hours of rehabilitation services (28). Indeed, compared with ordinary mental health treatments, the patients’ empowerment aimed at enhancing the individual’s independence and ability to self-manage the process of care contributed to the reduction of redundancies and omissions, producing higher treatment efficiency who, in turn, were likely to express fewer health needs (30).

##### Phenotype assessment of HB patient

Fontecedro et al. (49) analyzed the use of the individual HB in patients with severe clinical and social problems. They were also at higher risk than controls of aggressive or agitated behaviors, hallucinations and delusions, impairment in everyday life activities, and hospitalization.

##### Satisfaction of users

Most of the studies found medium–high levels of satisfaction with the HB programs among users and carers (30, 31, 46, 48). For example, Croft and Parish (46) reported that the interviewed perceived value in maintaining therapeutic relationships with providers over time and benefitted from providers’ availability. Welch et al. (42) reported that the PHB holders perceived improved in relationships between budget holders and their care staff as an opportunity given by the HB program. Furthermore, Larkin (43) interviewed carers who perceived enhancement of the carer–service user relationship, felt happier and healthier, and had more control over their lives.

**TABLE 3:** Findings’ summary in relation to existing knowledge on PB.

Domain	Existing knowledge (14)	New knowledge added
Choice and control of care and support	*Patient center-care* 1. Increased levels of perceived choice and control (56–58), confidence, independence, and power (56–59). Except for Cheshire West and Chester Council (2010): mental health service users felt less in control of their care and support 2. Increase sense of choice, flexibility with how time and resources were spent (58) and availability of services to budget holders (59). Except for Spandler and Vick (58): feelings of uncertainty particularly in patients that found difficult to articulate their needs	*Patient center-care* 1. Significant outcomes in terms of users’ health status improvement, personalization of interventions, user satisfaction, increase in autonomy, self-esteem, competence, coping mastery, autonomy support, choice and control, control over service choices, and cost savings (30, 31, 36, 37, 40–42) 2. Control over carers’ daily lives, quality of life, health, and well-being (43) 3. Power and control perceived by the staff (44) 4. Support in meeting basic material needs help patients in achieving or setting their personal goals (41, 46) *Stakeholder engagement* 1. Stakeholder-driven investigations may mitigate the challenges of patient-centered comparative effectiveness research (32) *Involvement of cares or staff* 1. Importance of strong leadership among staff groups and of increasing power to service providers and commissioners (40, 42) 2. Need of additional training if enacting PHB (39) 3. In the management of conflicts are important “good” social work skills and practitioner sensitivity (47) 4. Importance of involving carers in the service user personalization processes and in support planning (47) 5. Involvement of carers in the managing of the PBs (42) 6. Trust in carers (48) *Timely and suitable access to treatment* 1. More timely and suitable health treatment (28, 30, 40), reducing omissions and redundancies (30) 2. No differences in residential days or emergency service hours between groups (28) *Phenotype assessment of HB patient* 1. HB used for patients with severe clinical and social problems (49) *Satisfaction of users* 1. Medium-high levels of satisfaction among users and carers (30, 31, 46, 48) *Challenges of users, carers, and professionals* 1. Patients perceived lack in the ability to manage the budget themselves, felt unable to cope with the monitoring requirements or perceived themselves to be too out of control in themselves to be able to act consistently and responsibly (44) 2. Carers perceived the process of negotiating budgets with practitioners and agencies as difficult and procedures seeming complex to manage (50) 3. Carers perceived less involvement in the service user’s care, and the administration of the PB as stressful (43) 4. Key informants perceived the development of HBs as complex (39) 5. Nurses perceived the HB as a distraction from the work of nursing (45) 6. Social workers reported insufficient time to engage with the process of HB (45) 7. “When” to offer a PHB as often clients were referred at “crisis point” and what support could be purchased from the PHB (42) 8. HB perceived difficult to manage and the staff as an obstruction to the PB (44) 9. No easy to adjust to the opportunity to think and take responsibility for themselves (51) 10. Confusion and frustration at what patients saw as inconsistent policies (46) 11. Carers perceived the HB as a process not well designed for people with severe mental health conditions to manage, the “paperwork” as stressful, and the staff and local authorities’ processes management (43, 50) 12. More guidance was required to identify goals and make use of the PB to take a more active part in the society (42, 52) 13. Professionals perceived stressful to manage the problems they had experienced with the support provided by their local authorities (43), and balancing limited authority resources, budgets and staffing levels with requirements to meet carers’ identified needs and/or support expectations (47)
Impact on life	*Clinical improvement* 1. Benefits for mental health (57–59) and one had mixed findings (12) *Quality of life* 1. Improved quality of life/overall satisfaction (5, 60–62) 2. Improved goal achievement (59) 3. Greater sense of hope and recovery (58, 63) *Improvement in everyday life activities* Improved community participation (56, 61, 64) Improved physical health (57, 62, 64) Better relationships with people (57, 58), though this was not the experience of all participants (58) *Engagement in work/independent housing* 1. Keeping paid work (65), but another study showed no impact on employment (57)	*Clinical improvement* 1. Clinical improvement (30, 31, 33–35) 2. Positive outcomes (e.g., mental health and wellbeing, psychological benefits, social participation and relationships, and confidence) (31, 40, 41, 52) *Quality of life* 1. Better quality of life after obtaining a competitive employment 2. Better quality of life after access to economic capital (51) 3. Support in terms of how live patients’ lives (51) *Improvement in everyday life activities* 1. Purchases related to transportation and dentistry (27) 2. Benefits of leisure activity or running errands (48) 3. Request of independent engagement in healthy lifestyles (e.g., gym memberships), public transportation arts and culture, higher education, and informal education (53) 4. Key factors that enabled (familiar staff, preparation for planning alternative tomorrows with hope, communication and sharing information, family involvement, and activity planning) and challenged (unfamiliar staff, staffing resources, access to transport, and changing health status) successful implementation of community participation goals in person-centered planning 5. To achieve recovery goals, engagement in developing skills and/or knowledge, purchasing equipment (e.g., computer, camera, and TV), joining a group for social, health and fitness, and recreational purposes, and developing aspects of “the self” (40) 6. Purchases help to make progress toward financial support and physical well-being (41) *Engagement in work/independent housing* 1. Satisfaction or improvement or maintaining engagement in paid/competitive work (27) 2. Satisfaction or improvement or maintaining independent living (27) 3. Boost the shift toward personalized supported housing (36)
Service Use	*Frequency and quality of service use* 1. No study reported an increase in the use of inpatient services 2. Decrease in community mental health service use (6, 12, 13, 58, 61)	*Frequency and quality of service use* 1. No difference in service utilization between patient-centered medical home (PCMH) care and non-PCMH care or usual care (26) 2. Significant changes in the way resources were used and in the personalized intervention approach (36) *Lack of transitional support* 1. No solution for the gap between secondary school and adult life after high school (48)
Economic evaluations	1. PHBs are cost-effective (6) 2. Individual budgets are cost-neutral (5)	1. Reduced cases of institutionalization; higher appropriateness of health care services with consistent cost savings (30) 2. Financial relieve on the family (48) 3. Individualized funds useful to purchase activities for the consumers’ recovery (40) 4. No differences in terms of expenditures (26, 29) 5. Financial concerns with the monthly allowance amount left after paying attendants (48)

##### Challenges of users, carers, and professionals

Hamilton et al. (44) showed that some service users felt unsatisfied with HB because they believed that they could not manage the budget themselves, felt unable to cope with the monitoring requirements, or perceived themselves to be too out of control in themselves to act consistently responsibly.

Also, carers found the process of negotiating budgets with practitioners and agencies to be difficult and procedures seeming complex to manage (50), less involvement in the service user’s care, and perceived the administration of the PB as stressful (43). Also, key informants perceived the development of HBs as complex (39).

Some mental health nurses interviewed by Hamilton et al. (45) reported that the PB was perceived as a distraction from nursing work. Other nurses argued that specific skills that characterized their profession were being side-lined to engage in implementing PBs. Several social workers reported the perception of insufficient time to engage with the process of PB, perceiving it as bureaucratic, complicated, and time-consuming. However, social workers identified the voluntary or third sector as a potential resource for PB (45). The frontline staff interviewed by Welch et al. (42) perceived a challenge “when” to offer a PHB as often clients were referred at “crisis point” and what support could be purchased from the PHB.

Several studies found that HB may be stressful for users, carers, and professionals. For example, the PHB was perceived by users as difficult to manage and the staff as an obstruction to the PB (44). Other participants did not find it easy to adjust to the opportunity to think and take responsibility for themselves (51) and expressed confusion and frustration at what they saw as inconsistent policies, particularly for purchases that address basic needs like rent, transportation, and household goods (46).

Carers perceived the HB as a process not well designed for people with severe mental health conditions to manage, the “paperwork” as stressful, and the staff and local authorities’ processes management (43, 50). Larsen et al. (52) and Welch et al. (42) showed that more guidance was required to identify goals and use the PB to take a more active part in society.

Professionals perceived particularly stressful to manage the problems they had experienced with the support provided by their local authorities (43), and balancing limited authority resources, budgets, and staffing levels with requirements to meet carers’ identified needs and/or support expectations (47).

#### Impact on life

##### Clinical improvement

Most of the studies reported clinical improvement in patients involved in HB programs. Adinolfi et al. (30) reported improvement in patients with problems related to alcohol and/or drug addiction, cognitive, physical, or disability problems; problems associated with hallucinations and delusions, with depressed mood; mental and behavioral problems; and problems with relationships, with activities of daily living, with living conditions, and with occupation and activities. In addition, the (34, 35) showed improvement in patients using PHB along 2-year follow-up using validated tools (i.e., Health of Nation Outcome Scales—HoNOS), Brief Psychiatric Rating Scale—BPRS, and Global Assessment of Functioning—GAF). Most participants from Larsen et al. (52) and Peterson et al. (40) studies identified positive outcomes (e.g., mental health and wellbeing, psychological benefits, social participation and relationships, and confidence) from using personal-centered services. Leuci et al. (33) showed that patients enrolled in the PHB intervention, at the end of the 2-year follow-up, reported higher improvements in negative symptoms, disorganization, and activation dimension (including manic features and hyperactivity) and in HoNOS “Impairment” subscale. In addition, in the period between T1 and T2, patients enrolled in the PHB showed a further significant decrease in positive symptoms, activation and affective dimension (including depressive characteristics, suicidality, and anxiety), improvements in functioning, and in HoNOS “Impairment,” “Psychiatric Symptoms,” and “Behavioural Problems” subscales. Finally, Cook et al. (31) showed that the intervention users had significantly lower somatic symptom severity over time compared to the control group.

##### Quality of life

Several participants from the Tew et al. (51) reported that access to economic capital enabled them to have a better quality of day-to-day life. Others saw their budget as a mechanism for organizing personal support in terms of how they were living their lives (51).

##### Improvement in everyday life activities

Several studies found improvement in the everyday life activities of patients involved in HB. For example, Croft et al. (27) showed that most purchases were related to transportation and dentistry. Nine representatives of the Harry et al. (48) study discussed the benefits of having a care attendant that could take young adults into the community, whether doing a leisure activity or running errands. Snethen et al. (53) participants requested items that facilitated independent engagement in healthy lifestyles (e.g., gym memberships) and public transportation. Fewer participants requested arts and culture, higher education, and informal education. Finally, the majority of Peterson et al. (40) study participants, to achieve recovery goals, engaged in developing skills and/or knowledge, purchasing equipment (e.g., computer, camera, and TV), joining a group for social, health and fitness, and recreational purposes, and developing aspects of “the self.” Participants of the Spaulding-Givens et al. (41) study felt their purchases help them to make progress toward financial support and physical wellbeing.

##### Engagement in work/independent housing

Two studies showed satisfaction or improvement or maintaining engagement in paid/competitive work (27) and independent living (27) during the HB programs. Ridente and Mezzina (36) showed that the individual HB method boosted the shift toward personalized supported housing for people with severe mental health conditions and complex problems.

#### Service use

##### Frequency and quality of service use

On the one hand, Bowdoin et al. (26) showed that service utilization did not differ between participants who received PCMH care and participants who received non-PCMH care or usual care. On the other hand, Ridente and Mezzina (36) showed that the use of HB method brought about significant changes is the way resources were used and in the personalized intervention approach within local Mental Health Department teams (transparency, clarity on spending decisions, and increased awareness of the importance of rational use of resources based on an adequate turnover of projects).

##### Lack of transitional support

Two interviewed by Harry et al. (48) reported that the Cash and Counseling-based self-directed services program was not used for bridging the gap between secondary school and adult life after high school (i.e., they stayed at home for one or 2 years after high school).

#### Economic evaluations

On the one hand, Adinolfi et al. (30) observed that HB often led to reducing institutionalization and brings to higher appropriateness of healthcare services with consistent cost savings, two interviewed by Harry et al. (48) reported financial relief on the family, and Peterson et al. (40) showed that individualized funds were useful to purchase activities for the consumers’ recovery.

On the other hand, Bowdoin et al. (26) found no differences in terms of expenditures (sum of direct payments for medical care) between participants who received PCMH care and participants who received non-PCMH care or usual care. Croft et al. (29) showed no differences in the total service costs before and after program participation. Five representatives interviewed by Harry et al. (48) described financial concerns with the monthly allowance amount left after paying attendants.

## Discussion

The present systematic review has updated the existing literature since 2013 (14). More than 9,000 publications were screened. The scientific literature on PBs in the last 9 years is significantly increased (Table 3), and many valuable results have been achieved toward the definition of benefits and challenges of PBs for people with mental health conditions.

The studies have been conducted in the United Kingdom (*n* = 11), United States (*n* = 11), Italy (*n* = 6), and one in Australia. The majority of the studies have been conducted using qualitative methodologies (*n* = 20) based on not validated qualitative study-specific interviews. Only eight studies used validated tools to assess outcomes. One study did not specify the methodology used to assess the outcomes. In addition, the PBs’ outcomes were explored for the most in adult populations (*n* = 26). Many of the studies (*n* = 7) did not provide the age of the population interviewed. More than the 50% of females were enrolled in the majority of the studies’ samples (*n* = 20).

The studies included in the present systematic review show some limitations that make difficult in generalizing the results. First, the distribution of the countries among the included studies is only representative of the state of art in the western world. Also, comparison of PBs among countries is complicated because of the profoundly different characteristics of the healthcare system of reference. Future research should seek to depict the situation of the PB for people with mental health conditions in the rest of the world and describe in detail the characteristics of the healthcare system. Second, many studies included a small sample size, were mostly qualitative design, and did not use validated instruments to measure the outcomes, and the studies’ quality measured by the risk of bias was not always satisfactory. As Webber et al. (14) registered in their review, we also reported that most of the studies lacked justification for the research design and insufficient data analysis rigor. All qualitative studies lacked critical consideration of the researcher’s role, potential bias, and influence. All cross-sectional studies lacked the application of strategies to deal with potential confounding factors. Future cohort studies should seek to consider strategies to deal with confounding factors, strengthen the association between exposure and outcome, complete large time-span follow-ups, and recruit more homogenous experimental and control samples. More evidence-based research practice, longitudinal studies, and good quality studies using validated tools to assess the outcomes need to be conducted to explore in a more systematic manner the outcomes of the PBs. Third, the samples of the included studies were not representative of targeted population and lacked a full description of the phenotype of the participants with mental health conditions. For example, several studies did not describe the severity of the users’ symptoms, if users have intellectual disabilities, communication, or language problems. Finally, the payments used in the PB were often omitted. Those are fundamental aspects to consider when users are called to manage their PBs and should be carefully considered in future research. Also, future studies should seek to explore how and whether the personal PBs may be beneficial for target populations such as women, children, adolescents, elderly, and people with mental health conditions in comorbidity with intellectual disabilities. Fourth, as Webber et al. (14) observed in the studies older than 2013, interpretation is limited by the heterogeneity of the PB protocols: different payment, support, funding mechanisms, outcome measures, and context were described in each study. It urges to conduct large number of studies to be able to combine similar protocols for improving the outcomes’ interpretation.

Keeping into consideration these limitations, we can draw some general lessons. Positive outcomes for patients with mental health conditions utilizing PBs have been confirmed in terms of choice and control. The use of PB for people with mental health conditions showed several benefits in patient empowerment, stakeholder engagement, involvement of carers or staff in the PBs, and timely and suitable access to treatment. In general, several studies showed satisfaction for the PBs (30, 46, 48). Also, PBs improved users’ clinical outcomes, quality of life, and engagement in paid work and independent living. PBs brought to significant changes in the way resources were used and in the personalized intervention approaches (36, 40). PBs brought also to cost savings for the families (48) and the national health systems (30).

Concerns and challenges have been expressed on PBs in several studies. Users and carers perceived difficult and stressful the management and procedures of PBs (39, 43, 44, 46, 50, 51). Carers perceived difficulties in negotiating PBs with professionals (50) and felt less involvement in the care of their beloved ones (43). Professionals perceived the management of PBs as an additional burden in their work (43, 45, 47). Results on change in the frequency of the services’ use, efficacy of the services, and cost savings for the families or healthcare systems are inconsistent. On the one hand, some studies showed no differences in the frequency of service utilization, no solution for the gap in transition services (26, 48), no differences in terms of expenditures (26), and concerns with allowance (48). On the other hand, other studies showed that PBs led to cost savings for the healthcare system (30, 36) and the families (48). Studies that explore potential cost savings led by PBs should be increased in terms of quantity and quality.

The studies included in the present systematic review explored the outcomes of follow-ups longer than 2 years [except for one study Adinolfi et al. (30): 1 year], and two studies had follow-ups even longer [4, 8 years: (27); 10 years: (36)]. A positive aspect is that the recent studies we included in this work extended the time of the follow-ups compared to the studies in Webber et al. conducted before 2013 [follow-ups of 1 year or even less, (14)].

## Conclusion

Individuals with mental health conditions and/or with ID have extremely heterogeneous interests and needs that mental health services could address whether users and carers would have the possibility to self-direct their care (53). Being in charge of their own care, being able to express and implement their choice and control in their process of care, and jointly sharing the process management with carers and professionals showed improvement in responsibility and awareness, quality of life, independent living, paid work, clinical, psychological and social domains, and everyday aspects of the users’ and their carers’ life. However, the present systematic review showed that several challenges and concerns arise from the application of PBs and highlight the need to make the management of PBs less stressful and burdensome for users, carers, and professionals.

## Data availability statement

The original contributions presented in this study are included in the article/Supplementary material, further inquiries can be directed to the corresponding author.

## Author contributions

MS: conception. MS, MM, FF, LG, AC, EF, and GC: design of the work and interpretation of data. MM, FF, LG, AC, EF, and GC: data acquisition. MM, LG, EF, and GC: writing—original draft preparation. MS, MF, and GR: funding acquisition. All authors contributed to the writing—review and editing, and read and agreed to the published version of the manuscript.

## Footnotes

1
www.crd.york.ac.uk/prospero/

## References

[1] World Health Organization. *Regional office for the western pacific. People-centred health care: A policy framework.* Manila: World Health Organization, Western Pacific Region (2007).

[2] Department of Health. *Transforming adult social care. Local authority circular.* London: Crown Copyright (2008).

[3] Glasby J Littlechild R Glasby J. *Direct payments and personal budgets: Putting personalisation into practice.* 2nd ed. Bristol: Policy Press (2009). p. 217

[4] Gadsby EW. *Personal budgets and health: A review of the evidence. Policy research unit in commissioning and the healthcare system.* Canterbury: Centre for Health Services Studies, Kent (2013). p. 44

[5] Glendinning C Challis D Fernández JL Jacobs S Jones K Knapp M *Evaluation of the individual budgets pilot programme. Summary report.* York: University of York, Social Policy Research Unit (2008). p. 61

[6] Forder J Jones K Glendinning C Caiels J Welch E Baxter K *Evaluation of the personal health budget pilot programme.* Canterbury: University of Kent (2012).

[7] Pavolini E Ranci C. Restructuring the welfare state: Reforms in long-term care in Western European countries. *J Eur Soc Policy.* (2008) 18:246–59. 10.1177/0958928708091058

[8] The Health Foundation. *Personal health budgets.* London: The Health Foundation (2010).

[9] Davey V Fernández JL Knapp M Jolly D Swift P Tobin R *Direct payments: A national survey of direct payments policy and practice.* London: PSSRU (2007).

[10] Riddell S Pearson C Jolly D Barnes C Priestley M Mercer G. The development of direct payments in the UK: Implications for social justice. *Soc Policy Soc.* (2005) 4:75–85. 10.1017/S1474746404002209

[11] Alakeson V. *The contribution of self-direction to improving the quality of mental health services.* Washington, DC: U.S. Department of Health and Human Services (2007).

[12] Davidson J Baxter K Glendinning C Jones KC Forder JE Caiels J *Personal health budgets: Experiences and outcomes for budget holders at nine months. Fifth interim report.* Parassala: Department of Health (2012). p. 77

[13] Cook JA Russell C Grey DD Jonikas JA. Economic grand rounds: A self-directed care model for mental health recovery. *Psychiatr Serv.* (2008) 59:600–2. 10.1176/ps.2008.59.6.600 18511579

[14] Webber M Treacy S Carr S Clark M Parker G. The effectiveness of personal budgets for people with mental health problems: A systematic review. *J Ment Health.* (2014) 23:146–55. 10.3109/09638237.2014.910642 24803221

[15] Bhaumik S Tyrer FC McGrother C Ganghadaran SK. Psychiatric service use and psychiatric disorders in adults with intellectual disability. *J Intellect Disabil Res.* (2008) 52:986–95. 10.1111/j.1365-2788.2008.01124.x 19017168

[16] Sheehan R Hassiotis A Walters K Osborn D Strydom A Horsfall L. Mental illness, challenging behaviour, and psychotropic drug prescribing in people with intellectual disability: UK population based cohort study. *BMJ.* (2015) 351:h4326. 10.1136/bmj.h4326 26330451 PMC4556752

[17] Buckles J. A systematic review of the prevalence of psychiatric disorders in adults with intellectual disability, 2003–2010. *J Ment Health Res Intellect Disabil.* (2013) 6:181–207. 10.1080/19315864.2011.651682

[18] Castelpietra G Knudsen AKS Agardh EE Armocida B Beghi M Iburg KM The burden of mental disorders, substance use disorders and self-harm among young people in Europe, 1990–2019: Findings from the global burden of disease study 2019. *Lancet Regional Health Europe.* (2022) 16:100341. 10.1016/j.lanepe.2022.100341 35392452 PMC8980870

[19] Page MJ McKenzie JE Bossuyt PM Boutron I Hoffmann TC Mulrow CD The PRISMA 2020 statement: An updated guideline for reporting systematic reviews. *BMJ.* (2021) 372:n71. 10.1136/bmj.n71 33782057 PMC8005924

[20] Ouzzani M Hammady H Fedorowicz Z Elmagarmid A. Rayyan—a web and mobile app for systematic reviews. *Syst Rev.* (2016) 5:1–10. 10.1186/s13643-016-0384-4 27919275 PMC5139140

[21] Critical Appraisal Skills Programme. *CASP cohort study checklist.* (2018). Available online at: https://casp-uk.net/casp-tools-checklists/ (accessed January 7, 2021).

[22] Moola S Munn Z Tufanaru C Aromataris E Sears K Sfetcu R Chapter 7: Systematic reviews of etiology and risk. In: Aromataris E Munn Z editors. *Joanna briggs institute reviewer’s manual.* (Adelaide, SU: The Joanna Briggs Institute) (2017).

[23] Critical Appraisal Skills Programme. *CASP qualitative study checklist.* (2019). Available online at: https://casp-uk.net/casp-tools-checklists/ (accessed January 7, 2021).

[24] Critical Appraisal Skills Programme. *CASP randomised controlled trial standard checklist.* (2019). Available online at: https://casp-uk.net/casp-tools-checklists/ (accessed January 7, 2021).

[25] Tufanaru C Munn Z Aromataris E Campbell J Hopp L. Chapter 3: Systematic reviews of effectiveness. In: Aromataris E Munn Z editors. *Joanna briggs institute reviewer’s manual.* (Adelaide, SA: The Joanna Briggs Institute) (2017).

[26] Bowdoin JJ Rodriguez-Monguio R Puleo E Keller D Roche J. The patient-centered medical home model: Healthcare services utilization and cost for non-elderly adults with mental illness. *J Ment Health.* (2018) 27:574–82. 10.1080/09638237.2017.1385744 28990831

[27] Croft B İsvan N Parish SL Mahoney KJ. Housing and employment outcomes for mental health self-direction participants. *Psychiatr Serv.* (2018) 69:819–25. 10.1176/appi.ps.201700057 29759056 PMC6157604

[28] Croft B Battis K Isvan N Mahoney KJ. Service utilization before and after self-direction: A quasi-experimental difference-in-differences analysis of Utah’s mental health access to recovery program. *Adm Policy Ment Health.* (2019) 47:36–46. 10.1007/s10488-019-00969-4 31468285

[29] Croft B Battis K Ostrow L Salzer MS. Service costs and mental health self-direction: Findings from consumer recovery investment fund self-directed care. *Psychiatr Rehabil J.* (2019) 42:401–6. 10.1037/prj0000374 31070443

[30] Adinolfi P Starace F Palumbo R. Health outcomes and patient empowerment: The case of health budgets in Italy. *J Healthc Manag.* (2016) 18:117–33. 10.1177/0972063415625524

[31] Cook JA Shore S Burke-Miller JK Jonikas JA Hamilton M Ruckdeschel B Mental health self-directed care financing: Efficacy in improving outcomes and controlling costs for adults with serious mental illness. *Psychiatr Serv.* (2019) 70:191–201. 10.1176/appi.ps.201800337 30630401

[32] Kogan JN Schuster J Nikolajski C Schake P Carney T Morton SC Challenges encountered in the conduct of optimal health: A patient-centered comparative effectiveness study of interventions for adults with serious mental illness. *Clin Trials.* (2017) 14:5–16. 10.1177/1740774516670895 27681658

[33] Leuci E Pelizza L Landi G Quattrone E Maestri D Azzali S Personal health budget in patients with first episode psychosis: A new rehabilitation model based on a community care system in Italy. *Early Interv Psychiatry.* (2021) 16:221–30. 10.1111/eip.13145 33754490

[34] Pelizza L Leuci E Landi G Quattrone E Azzali S Pelosi A The “personal health budget” intervention model in early psychosis: Preliminary findings from the Parma experience. *Psychopathology.* (2020) 26:209–17. 10.36148/2284-0249-359

[35] Pelizza L Leuci E Landi G Maestri D Paulillo G Ceroni P Personal health budget as a new rehabilitation model for severe mental illness within a caring community: An Italian evaluation study of beneficial effects. *Aust N Z J Psychiatry.* (2020) 55:602–12. 10.1177/0004867420968918 33111536

[36] Ridente P Mezzina R. From residential facilities to supported housing: The personal health budget model as a form of coproduction. *Int J Ment Health.* (2016) 45:59–70. 10.1080/00207411.2016.1146510

[37] Thomas EC Zisman-Ilani Y Salzer MS. Self-determination and choice in mental health: Qualitative insights from a study of self-directed care. *Psychiatr Serv.* (2019) 70:801–7. 10.1176/appi.ps.201800544 31109262 PMC6718300

[38] Williams V Porter S. The meaning of ‘choice and control’for people with intellectual disabilities who are planning their social care and support. *J Appl Res Intellect Disabil.* (2017) 30:97–108. 10.1111/jar.12222 26500151

[39] Norrie C Woolham J Samsi K Manthorpe J. Skill mix: The potential for personal assistants to undertake health-related tasks for people with personal health budgets. *Health Soc Care Community* (2020) 28:922–31. 10.1111/hsc.12923 31854059

[40] Peterson S Buchanan A Falkmer T. The impact of services that offer individualised funds, shared management, person-centred relationships, and self-direction on the lived experiences of consumers with mental illness. *IJMHS.* (2014) 8:1–14. 10.1186/1752-4458-8-20 24944564 PMC4061914

[41] Spaulding-Givens J Hughes S Lacasse JR. Money matters: Participants’ purchasing experiences in a budget authority model of self-directed care. *Soc Work Ment Health.* (2019) 17:323–43. 10.1080/15332985.2018.1555105

[42] Welch E Jones K Caiels J Windle K Bass R. Implementing personal health budgets in England: A user-led approach to substance misuse. *Health Soc Care Community.* (2017) 25:1634–43. 10.1111/hsc.12396 27723160 PMC5573945

[43] Larkin M. Developing the knowledge base about carers and personalisation: Contributions made by an exploration of carers’ perspectives on personal budgets and the carer–service user relationship. *Health Soc Care Community.* (2015) 23:33–41. 10.1111/hsc.12131 25315982

[44] Hamilton S Tew J Szymczynska P Clewett N Manthorpe J Larsen J Power, choice and control: How do personal budgets affect the experiences of people with mental health problems and their relationships with social workers and other practitioners? *Br J Soc Work.* (2015) 46:719–36. 10.1093/bjsw/bcv023

[45] Hamilton S Manthorpe J Szymczynska P Clewett N Larsen J Pinfold V Implementing personalisation in integrated mental health teams in England. *J Interprof Care.* (2015) 29:488–93. 10.3109/13561820.2015.1035777 26171867

[46] Croft B Parish S. Participants’ assessment of the impact of behavioral health self-direction on recovery. *Community Ment.* (2016) 52:781–92. 10.1007/s10597-016-9999-0 26911369 PMC4996770

[47] Mitchell W Brooks J Glendinning C. Carers’ roles in personal budgets: Tensions and dilemmas in front line practice. *Br J Soc Work.* (2015) 45:1433–50. 10.1093/bjsw/bcu018

[48] Harry ML MacDonald L McLuckie A Battista C Mahoney EK Mahoney KJ. Long-term experiences in cash and counseling for young adults with intellectual disabilities: Familial programme representative descriptions. *J Appl Res Intellect Disabil.* (2017) 30:573–83. 10.1111/jar.12251 26892813

[49] Fontecedro E Furlan M Tossut D Pascolo-Fabrici E Balestrieri M Salvador-Carulla L Individual health budgets in mental health: Results of its implementation in the Friuli Venezia Giulia region Italy. *Int J Environ.* (2020) 17:5017. 10.3390/ijerph17145017 32668599 PMC7400620

[50] Hamilton S Szymczynska P Clewett N Manthorpe J Tew J Larsen J The role of family carers in the use of personal budgets by people with mental health problems. *Health Soc Care Community.* (2015) 25:158–66. 10.1111/hsc.12286 26435491

[51] Tew J Larsen J Hamilton S Manthorpe J Clewett N Pinfold V ‘And the stuff that I’m able to achieve now is really amazing’: The potential of personal budgets as a mechanism for supporting recovery in mental health. *Br J Soc Work.* (2015) 45:i79–97. 10.1093/bjsw/bcv097

[52] Larsen J Tew J Hamilton S Manthorpe J Pinfold V Szymczynska P Outcomes from personal budgets in mental health: Service users’ experiences in three English local authorities. *J Ment Health.* (2015) 24:219–24. 10.3109/09638237.2015.1036971 26207416

[53] Snethen G Bilger A Maula EC Salzer MS. Exploring personal medicine as part of self-directed care: Expanding perspectives on medical necessity. *Psychiatr Serv.* (2016) 67:883–9. 10.1176/appi.ps.201500311 27032663

[54] Hitchen S Williamson GR Watkins M. Personal budgets for all? Implementing self-directed support in mental health services. *Action Res.* (2015) 13:372–91. 10.1177/1476750314568207

[55] Veltro F Morosini P Gigantesco A Casacchia M Roncone R Dell’Acqua G A new self-report questionnaire called” ABC” to evaluate in a clinical practice the aid perceived from services by relatives, needs and family burden of severe mental illness. *Clin Pract Epidemiol Ment Health.* (2007) 3:1–6. 10.1186/1745-0179-3-15 17877813 PMC2031885

[56] Eost-Telling C. *stockport self directed support pilot in mental health: Final Report of the evaluation of the self directed support pilot.* Chester: University of Chester (2010).

[57] Hatton C Waters J. *The national personal budget survey.* Lancaster, PA: Lancaster University (2011).

[58] Spandler H Vick N. *Direct payments, independent living and mental health: An evaluation.* London: Health and Social Care Advisory Service (2004). 10.1111/j.1365-2524.2006.00598.x 16460360

[59] Teague GB Boaz TL. *Evaluation of the adult mental health self-directed care project.* Tallahassee, FL: Florida Department of Children and Families (2003).

[60] Homer T Gilder P. *A review of self-directed support in Scotland.* Edinburgh: Scottish Government Social Research (2008).

[61] Rogers L. *Self directed support for mental health service users in west Sussex.* West Sussex: West Sussex County Council (2009).

[62] Shen C Smyer M Mahoney KJ Simon-Rusinowitz L Shinogle J Norstrand J Consumer-directed care for beneficiaries with mental illness: Lessons from New Jersey’s Cash and counseling program. *Psychiatr Serv.* (2008) 59:1299–306. 10.1176/ps.2008.59.11.1299 18971406

[63] Coyle D. *Recovery budgets in a mental health service: Evaluating recovery budgets for people accessing an early intervention service and the impact of working with self directed services on the team members within a North west of England NHS trust.* Chester: University of Chester (2009).

[64] Lawson S Pearmain G Waters J. *Finding our way: The story of self-directed support in Barnsley.* West Midlands: In control (2010).

[65] Cheshire West and Chester Council. *Findings from the personal budgets survey.* Cheshire: Cheshire West & Chester Council (2010).

