# Supplementary material for: Benefits and challenges of a personal budget for people with mental health conditions or intellectual disability: A systematic review
Source: Front Psychiatry. 2022 Aug 4;13:974621. doi: 10.3389/fpsyt.2022.974621 (PMC9386381; doi:10.3389/fpsyt.2022.974621)
Supplement: Supplementary file 1 [file Data_Sheet_1.docx]

Supplementary Material

1. **Supplementary Table 1.** The string focused on Population, Intervention, Comparison, and Outcomes (PICO) for PubMed and PsycINFO.

| **Domain** | **Search strategy** |
| --- | --- |
| **Population** | "mental health" OR exp mental disorders OR "intellectual* disab*" OR "intellectual* disorder*" OR "intellectual* impair*" OR "mental* retard*" OR "mental* challenged" OR "mental* handicap*" OR "mental* impair*" OR "mental* deficien*" OR "learning disab*" OR "learning disorder*" OR "learning impair*" OR "development* disab*" OR "development* disorder*" OR "development* impair*" OR "subaverage intelligence" |
| **Intervention** | "cash counselling" OR "cash for care" OR "consumer directed care" OR "consumer-directed care" OR "direct payment*" OR "indicative allocation" OR "individual budget*" OR "individual service fund*" OR "individual* service* design*" OR "managed account*" OR "managed budget*" OR "notional budget*" OR "personal budget*" OR "personal health budget*" OR personalization OR personalisation OR "personalised care" OR "person centred" OR "person-centred" OR "person-centered" OR "person centered" OR "pooled budget*" OR "recovery budget*" OR "resource allocation system" OR "self directed assessment*" OR "self-directed assessment*" OR "self-directed care" OR "self directed care" OR "self-directed support*" OR "self directed support*" OR "support plan*" OR "virtual budget*" OR "health management" OR "patient empowerment" OR "co-production of care" OR "self managed care" OR "self-managed care" OR "individualized funding" OR "lifestyle plan*" OR "essential lifestyle* plan*" OR "personal future* plan*" OR "future* plan*" OR "shared action* plan*" OR "care management" |
| **Comparison** | Not applicable |
| **Outcome** | Desired and undesirable effects of Personal Budget |
| **Limits** | Humans; since August 2014; English |

1. **Supplementary Table 2.** Quality assessment score of the included studies using the CASP Qualitative Study Checklist.

| **Study** |  | **Items** | | | | | | | | | |  | **Score** |
| --- | --- | --- | --- | --- | --- | --- | --- | --- | --- | --- | --- | --- | --- |
|  |  | 1 | 2 | 3 | 4 | 5 | 6 | 7 | 8 | 9 | 10 |  |  |
| Adinolfi, 2016 |  | + | + | - | - | - | - | - | - | + | + |  | 4/10 |
| Cook, 2019 |  | + | + | - | + | + | + | - | + | + | + |  | 8/10 |
| Croft, 2016 |  | + | - | - | + | + | - | + | + | + | + |  | 7/10 |
| Hamilton, 2015a |  | + | + | - | + | - | - | - | - | - | + |  | 4/10 |
| Hamilton, 2015b |  | + | + | - | + | - | - | + | + | - | + |  | 6/10 |
| Hamilton, 2016 |  | + | - | - | + | - | - | + | - | - | + |  | 4/10 |
| Harry, 2016 |  | + | + | - | - | + | - | - | - | - | - |  | 3/10 |
| Hitchen, 2015 |  | + | - | - | - | + | - | - | - | - | + |  | 3/10 |
| Kogan, 2016 |  | + | - | - | + | + | - | + | - | - | - |  | 4/10 |
| Larkin, 2015 |  | + | + | + | + | + | - | + | - | + | + |  | 8/10 |
| Larsen, 2015 |  | + | + | + | + | + | - | + | + | + | + |  | 9/10 |
| Mitchell, 2014 |  | + | + | - | - | + | - | + | - | - | + |  | 5/10 |
| Norrie, 2020 |  | + | + | + | + | + | - | + | - | + | + |  | 8/10 |
| Peterson, 2014 |  | + | + | + | + | + | - | + | + | - | + |  | 8/10 |
| Ridente, 2016 |  | + | - | - | - | - | - | - | - | + | + |  | 3/10 |
| Spaulding-Givens,2018 |  | + | + | - | + | + | - | - | + | + | + |  | 7/10 |
| Tew, 2015 |  | + | + | + | - | - | - | + | - | - | + |  | 5/10 |
| Thomas,2019 |  | + | + | - | + | - | - | - | + | - | + |  | 5/10 |
| Welch, 2016 |  | + | + | - | - | + | - | + | - | + | + |  | 6/10 |
| Williams et al., 2015 |  | + | + | + | - | + | - | + | - | - | + |  | 6/10 |

*Note.* Scoring was coded as ‘Yes’ (+), ‘No’ (–). Item 1 = Clear statement of aim; Item 2 = Appropriate qualitative methodology; Item 3 = Appropriate research design; Item 4 = Appropriate recruitment strategy; Item 5 = Data collection addresses research issue; Item 6 = Relationship between researcher and participants considered; Item 7 = Ethical consideration; Item 8 = Appropriate data analysis; Item 9 = Clear statement of findings; Item 10 = Research value (Critical Appraisal Skills Programme. CASP Qualitative Study Checklist, 2019).

1. **Supplementary Table 3.** Quality assessment score of the included studies using the JBI Critical Appraisal Checklist for Cross Sectional Studies.

|  |  | **Items** | | | | | | | |  | **Score** |
| --- | --- | --- | --- | --- | --- | --- | --- | --- | --- | --- | --- |
|  |  | 1 | 2 | 3 | 4 | 5 | 6 | 7 | 8 |  |  |
| Fontecedro, 2020 |  | + | + | NA | + | + | - | + | + |  | 6/8 |
| Snethen, 2016 |  | + | + | + | - | + | - | + | + |  | 6/8 |

*Note.* Scoring was coded as ‘Yes’ (+), ‘No’ (–), ‘Unclear’ (U), or ‘Not Applicable’ (NA). Item 1 = Clear definition of inclusion criteria; Item 2 = Study subjects and setting described in detail; Item 3 = Exposure measured in a valid and reliable way; Item 4 = Objective, standard criteria used for measurement of condition; Item 5 = Confounding factors identified; Item 6 = Statement of strategies to deal with confounding factors; Item 7 = Outcomes measured in a valid and reliable way; Item 8 = Appropriateness of statistical analysis (Moola et al., 2018).

1. **Supplementary Table 4.** Quality assessment score of the included studies using the CASP Cohort Study Checklist.

| **Study** |  | **Items** | | | | | | | | | | | | | |  | **Score** |
| --- | --- | --- | --- | --- | --- | --- | --- | --- | --- | --- | --- | --- | --- | --- | --- | --- | --- |
|  |  | 1 | 2 | 3 | 4 | 5a | 5b | 6a | 6b | 7 | 8 | 9 | 10 | 11 | 12 |  |  |
| Leuci, 2021 |  | + | + | + | + | + | - | - | - | - | + | + | + | + | + |  | 10/14 |
| Pellizza, 2020a |  | + | + | + | + | + | - | - | - | - | + | + | + | + | + |  | 10/14 |
| Pellizza, 2020b |  | + | + | + | + | + | - | - | - | - | + | + | + | + | + |  | 10/14 |

*Note.* Scoring was coded as ‘Yes’ (+), ‘No’ (–), ‘Can’t tell’ (?). Item 1 = Clear focused issue; Item 2 = Appropriate cohort recruitment; Item 3 = Exposure accurately measured; Item 4 = Outcome accurately measured; Item 5a = Identification of confounding factors; 5b = Consideration of confounding factors in design and/or analysis; Item 6a = Follow up complete; 6b = Follow up long enough; Item 7 = Strong exposure and outcome relation; Item 8 = Precis results; Item 9 = Believe the results; Item 10 = Applicability of results in local population; 11= Results fit with evidence; 12 = Implication for practice [Critical Appraisal](file:///C:\Users\gila_letizia\AppData\Local\Packages\Microsoft.MicrosoftEdge_8wekyb3d8bbwe\TempState\Downloads\Critical%20Appraisal) Skills Programme. CASP Cohort Study Checklist Checklist, 2019.

1. **Supplementary Table 5.** Quality assessment score of the included studies using the JBI Critical Appraisal Checklist for Quasi-Experimental Studies (non-randomized experimental studies).

|  |  | **Items** | | | | | | | | |  | **Score** |
| --- | --- | --- | --- | --- | --- | --- | --- | --- | --- | --- | --- | --- |
|  |  | 1 | 2 | 3 | 4 | 5 | 6 | 7 | 8 | 9 |  |  |
| Bowdoin, 2017 |  | + | - | - | + | - | + | + | + | + |  | 6/9 |
| Croft, 2018 |  | + | + | - | + | + | + | + | + | + |  | 8/9 |
| Croft, 2019a |  | + | + | - | + | + | - | + | - | + |  | 6/9 |
| Croft, 2019b |  | + | - | - | - | + | NA | NA | - | + |  | 3/9 |

*Note.* Scoring was coded as ‘Yes’ (+), ‘No’ (–), ‘Unclear’ (U), or ‘Not Applicable’ (NA). Item 1 = Clear ‘cause’ and ‘effect’; Item 2 = Participants included in any comparisons similar; Item 3 = Participants included in any comparisons received similar treatment/care, exposure/intervention; Item 4 = Presence of a control group; Item 5 = Multiple measurements of outcome pre and post intervention/exposure; Item 6 = Follow up complete, if not, differences between groups in terms of their follow up adequately described/analyzed; Item 7 = Outcomes included in any comparisons measured in the same way; Item 8 = Outcomes measured in a reliable way; Item 9 = Appropriateness of statistical analysis (Tufanaru ert al., 2017).

**Supplementary Figure 1.** PRISMA Checklist.

| **Section and Topic** | **Item #** | **Checklist item** | **Reported on page #** |
| --- | --- | --- | --- |
| **TITLE** | | |  |
| Title | 1 | Identify the report as a literature review. | 1 |
| **ABSTRACT** | | |  |
| Abstract | 2 | Provide a structured summary including, as applicable: background; objectives; data sources; study eligibility criteria, participants, and interventions; study appraisal and synthesis methods; results; limitations; conclusions and implications of key findings.  See the [PRISMA 2020 for Abstracts checklist](http://www.prisma-statement.org/Extensions/Abstracts.aspx) for the complete list. | 1 |
| **INTRODUCTION** | | |  |
| Rationale | 3 | Describe the rationale for the review in the context of existing knowledge, i.e., what is already known about your topic. | 2-6 |
| Objectives | 4 | Provide an explicit statement of the objective(s) or question(s) the review addresses with reference to participants, interventions, comparisons, outcomes, and study design (PICOS). | 5-6 |
| **METHODS** | | |  |
| Eligibility criteria | 5 | Specify the inclusion and exclusion criteria for the review and how studies were grouped for the syntheses with study characteristics (e.g., PICOS, length of follow-up) and report characteristics (e.g., years considered, language, publication status) used as criteria for eligibility, giving rationale. | 6-7 |
| Information sources | 6 | Specify all databases, registers, websites, organisations, reference lists and other sources searched or consulted to identify studies. Specify the date when each source was last searched or consulted. | 7 |
| Search strategy | 7 | Present the full search strategies for all databases, registers and websites, including any filters and limits used. | Supplementary Table 1 |
| Selection process | 8 | State the process for selecting studies (i.e., screening, eligibility).  Specify the methods used to decide whether a study met the inclusion criteria of the review, including how many reviewers screened each record and each report retrieved, whether they worked independently, and if applicable, details of automation tools used in the process. | 7-8 |
| Study risk of bias assessment | 11 | Specify the methods used to assess risk of bias in the included studies, including details of the tool(s) used, how many reviewers assessed each study and whether they worked independently, and if applicable, details of automation tools used in the process. | 9 |
| **RESULTS** | | |  |
| Study selection | 16a | Describe the results of the search and selection process, from the number of records identified in the search to the number of studies included in the review, ideally using a flow diagram. | 10 |
|  | 16b | Cite studies that might appear to meet the inclusion criteria, but which were excluded, and explain why they were excluded. | N/A |
| Study characteristics | 17 | Cite each included study and present its characteristics (e.g., study size, PICOS, follow-up period). | Table 1 |
| Risk of bias in studies | 18 | Present assessments of risk of bias for each included study. | Supplementary Table 2-5 |
| Results of individual studies | 19 | For all outcomes, present, for each study: (a) summary statistics for each group (where appropriate) and (b) an effect estimate and its precision (e.g. confidence/credible interval), ideally using structured tables or plots. | Table 2 |
| **DISCUSSION** | | |  |
| Discussion | 23a | Provide a general interpretation of the results in the context of other evidence. | 26-31; Table 3 |
|  | 23b | Discuss any limitations of the evidence included in the review. | 26-28 |
|  | 23c | Discuss any limitations of the review processes used. | N/A |
|  | 23d | Discuss implications of the results for practice, policy, and future research. | 28-31 |
| **OTHER INFORMATION** | | |  |
| Registration and protocol | 24a | Provide registration information for the review, including register name and registration number, or state that the review was not registered. | 6 |
|  | 24b | Indicate where the review protocol can be accessed, or state that a protocol was not prepared. | 6 |
|  | 24c | Describe and explain any amendments to information provided at registration or in the protocol. | N/A |
| Support | 25 | Describe sources of financial or non-financial support for the review, and the role of the funders or sponsors in the review. | 31 |
| Competing interests | 26 | Declare any competing interests of review authors. | 31 |
| Availability of data, code, and other materials | 27 | Report which of the following are publicly available and where they can be found: template data collection forms; data extracted from included studies; data used for all analyses; analytic code; any other materials used in the review. | N/A |

Source: Moher, D.; Liberati, A.; Tetzlaff, J.; Altman, D.G.; Altman, D.; Antes, G.; Atkins, D.; Barbour, V.; Barrowman, N.; Berlin, J.A.; et al. Preferred reporting items for systematic reviews and meta-analyses: The PRISMA statement.
